# To Ascertain the Purity of Chloroform

**Published:** 1862-12

**Authors:** 


*To ascertain the Purity of Chloroform.*—M. Heintz has stated that Chloroform is not attacked by sodium or potassium, even at the boiling point. M. Hardy proposes to employ this fact as a means of ascertaining the purity of Chloroform. If a small piece of sodium is thrown into pure Chloroform, no action whatever takes place; if, on the contrary, any impurity, such as alcohol, &c., &c., be present, then a disengagement of gas takes place.—*Pharmaceutical Journal.*

